# The Role of Olfactory Genes in the Expression of Rodent Paternal Care Behavior

**DOI:** 10.3390/genes11030292

**Published:** 2020-03-10

**Authors:** Tasmin L. Rymer

**Affiliations:** 1 College of Science and Engineering, James Cook University, P. O. Box 6811, Cairns, QLD 4870, Australia; tasmin.rymer@jcu.edu.au; Tel.: +61-7-4232-1629; 2 Centre for Tropical Environmental and Sustainability Sciences, James Cook University, P. O. Box 6811, Cairns, QLD 4870, Australia; 3 School of Animal, Plant and Environmental Sciences, University of the Witwatersrand, Private Bag 3, WITS, Johannesburg 2050, South Africa

**Keywords:** discrimination, main olfactory system, olfaction, paternal care, recognition, vomeronasal system

## Abstract

Olfaction is the dominant sensory modality in rodents, and is crucial for regulating social behaviors, including parental care. Paternal care is rare in rodents, but can have significant consequences for offspring fitness, suggesting a need to understand the factors that regulate its expression. Pup-related odor cues are critical for the onset and maintenance of paternal care. Here, I consider the role of olfaction in the expression of paternal care in rodents. The medial preoptic area shares neural projections with the olfactory and accessory olfactory bulbs, which are responsible for the interpretation of olfactory cues detected by the main olfactory and vomeronasal systems. The olfactory, trace amine, membrane-spanning 4-pass A, vomeronasal 1, vomeronasal 2 and formyl peptide receptors are all involved in olfactory detection. I highlight the roles that 10 olfactory genes play in the expression of direct paternal care behaviors, acknowledging that this list is not exhaustive. Many of these genes modulate parental aggression towards intruders, and facilitate the recognition and discrimination of pups in general. Much of our understanding comes from studies on non-naturally paternal laboratory rodents. Future studies should explore what role these genes play in the regulation and expression of paternal care in naturally biparental species.

## 1. Introduction

In order to survive, all animals must detect, interpret and respond to an array of sensory information in their immediate environment [1]. Animals need to locate and assess the quality of food, detect and avoid predators, and identify mates and competitors [1]. Animals can gain information about these resources or threats, as well as convey information to other individuals, via numerous modalities, including vision and olfaction. For many mammals, particularly rodents, olfaction is most likely the dominant sensory modality [2,3]. Social behaviors, including parent/offspring interactions, are also strongly regulated by olfactory cues (e.g., prairie voles (*Microtus ochrogaster*) [4] and Syrian hamsters (*Mesocricetus auratus*) [5]).

While mammalian maternal care is essential for offspring survival, mammalian paternal care is rare (5%–10% of species [6]). This is most likely because the costs associated with paternal care (e.g., predation risk [7], increased energetic expenditure [8], loss of mating opportunities [9], and reduced survival [10]), and the inability of males to physically associate with offspring during prenatal development [11], are major limiting factors in the evolution of paternal care [12]. However, paternal males can significantly influence offspring growth, survival, and cognitive and behavioral development [6]. Consequently, it is necessary to understand what factors play a role in the expression of paternal care when it does occur.

The ability to detect, recognize, and discriminate olfactory cues of social significance between fathers and offspring is dependent on complex neural mechanisms that may be regulated by specific olfactory genes. Here, I consider the role of olfaction in the expression of paternal care behavior in rodents. I first describe the behavioral machinery [13] of paternal care in general, and how this is connected with brain regions associated with the detection and interpretation of olfactory cues. I then discuss the genetic regulation of olfaction in the different olfactory systems in general. Finally, I discuss how olfactory cues might regulate the expression of paternal care behavior, highlighting the roles that 10 different olfactory genes may play in the expression of paternal care behavior. This list is not exhaustive, and there are likely multiple other genes that could be equally important. While indirect paternal care (e.g., alarm calling, [14]) is an important component of the paternal repertoire, I only focus on direct paternal care behavior (retrieval, huddling, nest building and grooming) because indirect paternal care does not require direct pup contact, whereas direct paternal care behavior does. I rely extensively on studies from laboratory mice, which are not naturally paternal, because the predominant literature on olfactory regulation of paternal care behavior comes from these studies, and the literature is decidedly depauperate on how the genetic mechanisms of olfaction moderate paternal care in biparental species. Nevertheless, these studies provide a starting point for those interested in mechanisms underlying the expression of paternal care in biparental species.

## 2. Neural Regulation of Paternal Care Behavior

### 2.1. Brain Regions Implicated in the Regulation and Expression of Paternal Care

The most important brain region associated with paternal care behavior is the hypothalamic medial preoptic area (MPOA [15,16]). The MPOA is anatomically connected to the bed nucleus of the stria terminalis (BNST) and the amygdala [17,18], as well as the lateral preoptic area (LPOA [19]), and the adjoining substantia innominata (SI [20]). Lateral efferent neurons project from the MPOA to the LPOA and SI, and pass through the lateral hypothalamus (LH) to the ventral tegmental area (VTA [19]). Neuronal disruption to the central MPOA and the lateral efferent neurons [21,22] can disrupt paternal care behavior, specifically retrieval behavior. Different subregions or neuron populations in the MPOA may affect an individual’s responsiveness to pup-specific odor cues [23].

The amygdala may also play an important role in the regulation of paternal care because of its distinctive neuronal heterogeneity, specifically, the caudal olfactory cortex [24]. The olfactory tubercle receives direct information from the main olfactory bulb (OB), whereas the medial amygdalar nucleus (meA), which lies adjacent to the olfactory tubercle, receives direct information from the accessory olfactory bulb (AOB [25]).

### 2.2. Olfactory Systems and Associations with Brain Regions Implicated in Paternal Care

Mammalian olfactory systems are complex, remarkably precise (one odorant receptor gene expressed per cell; [26]) and allow mammals to recognize and discriminate a large diversity of odorant molecules [27]. There are two main, anatomically and functionally distinct chemoreceptor systems [28], namely the main olfactory system (MOS) and the vomeronasal system (VNS). It has been suggested that the MOS primarily detects volatile odorants from the environment [29,30], whereas the VNS primarily detects non-volatile odorants from conspecifics [28,30,31], although both systems can, to a degree, detect both types of odor cues [30]. The Grueneberg ganglion, a chemosensory organ that appears to mediate behavioral responses to alarm pheromones in rodents [32], and the septal organ of Masera, a patch of sensory epithelium separate to the MOE that may have a dual role in surveying food or conspecific sexual odors [33], are not considered here.

#### 2.2.1. The Main Olfactory System (MOS)

**Figure 1:**
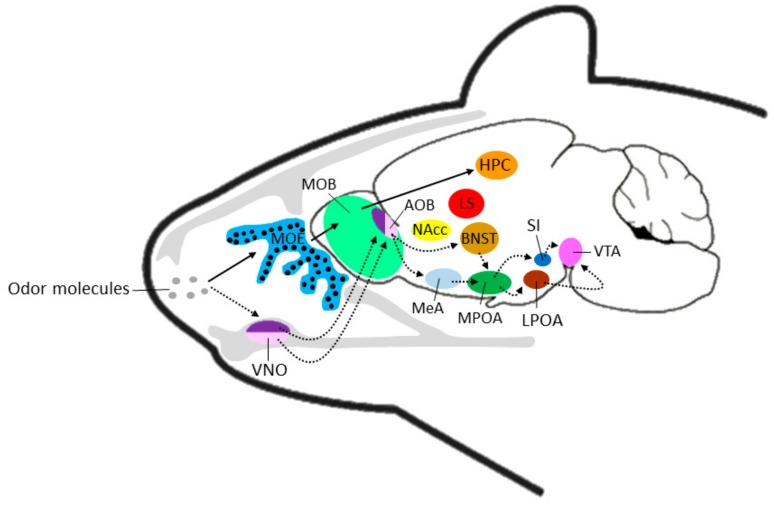
Schematic representation of transmission of olfactory information via the main olfactory system (solid black arrows) or the vomeronasal system (broken arrows) to corresponding brain regions. Black circles in the MOE indicate the broad localization of olfactory sensory neurons. Note: locations of brain regions not exact and for pictorial purposes only. AOB: accessory olfactory bulb; BNST: bed nucleus of the stria terminalis; HPC: hippocampus; LS: lateral septrum; MOB: main olfactory bulb; MOE: main olfactory epithelium; MeA: medial amydala; LPOA: lateral preoptic area; MPOA: medial preoptic area; NAcc: nucleus accumbens; SI: substantia innominata; VNO: vomeronasal organ; VTA: ventral tegmental area.

The nose houses the MOS, which consists of the main olfactory epithelium (MOE; Figure 1). This is the primary site for the detection of volatile odorants [1]. The main olfactory sensory neurons (OSNs) in the MOE, of which there are approximately 10 million in vertebrates, are located directly in the nasal airstream; thus, stimulus access simply requires passive respiration or a sniffing action [34]. The 500–1000 olfactory receptors (ORs [1,27,35]) that belong to the rhodopsin-like G-protein-coupled receptor superfamily (GPCRs [26,35,36]), are located in the cell membranes of the OSNs, and bind specific odorant ligands [35]. While the ORs are responsible for detecting chemosensory cues, they are also involved in axonal guidance to the brain [36,37].

Neurons that express the same OR type converge at similar sites (glomeruli) within the OB [38], forming synapses with mitral [36,38] or tufted cells [39] and conveying olfactory information from the MOE to the OB [40]. The signals from each OR are transferred to the anterior olfactory nucleus, the cortical amygdala [30] and a small number of pyramidal cells that form clusters in the piriform cortex (PC) of the olfactory cortex [41]. The PC cells project to the orbitofrontal cortex, amygdaloid cortex, prefrontal cortex, perirhinal cortex and entorhinal cortex, through which they access the hippocampus (Figure 1; [42]). Androgen and estrogen cellular receptors are expressed in the PC [43], suggesting a responsiveness to hormones that may regulate sexual and paternal behaviors [3]. Importantly, olfactory bulbectomy negatively affects paternal care in male prairie voles [44].

#### 2.2.2. The Vomeronasal System (VNS)

Closely associated with the MPOA is the accessory olfactory or VNS (Figure 1; [45]). The VNS is primarily involved with the reception and decoding of olfactory cues, providing a relatively direct pathway to the amygdala [46], BNST [39] and hypothalamic areas [47].

The vomeronasal organ (VNO; Figure 1) of the VNS is an extraordinarily sensitive structure [48]. In rodents, the morphological complexity of the VNO is greater than any other mammal [49]. The VNO detects both volatile and non-volatile olfactory signals [2], and neurons that express the same receptor form multiple glomeruli within the AOB [34]. Neural projections transfer the olfactory signals to several brain regions via the AOB [50], including the corticomedial amygdala [24] and the BNST (Figure 1; [51]). Projections from the AOB also extend to the medial (Figure 1) and posteromedial cortical (C3) amygdaloid nuclei [51] and the ventral hypothalamus [52]. Interestingly, there is sexual dimorphism in the AOB [53], meA [54] and BNST [54,55].

The MPOA then receives these impulses (Figure 1), activating Galanin-expressing neurons (MPOA^Gal^ [22]), and this cascade of impulses then activates neurons in the LPOA and SI (Figure 1; [20]). From here, LPOA descending efferent neurons, which are localized in the dorsal LH [19], trigger neurons in the VTA (Figure 1). The VTA is part of the dopaminergic reward system, and is associated with reinforcement learning, with unweaned offspring being a strong reinforcing stimulus to males [56]. This pathway likely influences the processing of pup-related olfactory cues, and mediates and regulates pup-directed aggression [57]. Disruptions to this pathway inhibit infanticide and promote paternal care [22,58].

## 3. Genetic Regulation of Olfaction

### 3.1. Genetic Regulation in the Main Olfactory System

Mammals can recognize and discriminate thousands of odor molecules due to a large multigene family (±1400 functional genes [29]) in the MOE that encodes the ORs. Each OSN expresses only one or a few odorant receptor genes [2,36,59,60], and the genes are randomly monoallelically expressed (i.e., half express the maternal allele while the other half express the paternal allele [36,59]). The ORs are bound to the G-protein *G*_α*olf*_ [30] and are typically not very selective. Thus, an OSN typically responds to a range of related odor cues (i.e., combinatorial in nature [61]). In mammals, ORs fall into two major groups (phylogenetic clades; [62]): "Class I" ORs (known as fish-like receptors as they were first identified in fish) comprise approximately 10% of functional ORs, while "Class II" ORs (mammalian-like receptors) comprise approximately 90% [40]. It is thought that a subset of some of these ORs from both classes, respond to volatile compounds in food, thereby influencing foraging behavior and food preferences [63].

In addition to the ORs, trace amine receptors (TAARs) are also expressed by neurons localized in the MOE [63,64,65], and are activated by distinct combinations of volatile amines, many of which occur in urine [66]. There are between 15 and 17 TAARs found in rodents [64,66]. TAARs are expressed in a small number of OSNs [66] and, like ORs, they are expressed in a mutually exclusive manner [64]. Interestingly, TAARs in the MOE are localized to G_αolf_–expressing sensory neurons that can stimulate cyclic adenosine monophosphate (cAMP) pathways, indicating that they couple to canonical olfactory pathways [66]. In rodents, G_αolf_ is highly expressed in the medium spiny neurons of the striatum, which houses the dopamine 1 receptor [67] and is critical for transduction of the ORs and complete olfactory function [68].

Lastly, a set of molecularly atypical neurons residing in the MOE expresses other non-GPCR receptors (guanylate cyclase GC-D) that are encoded by membrane-spanning 4-pass A (Ms4a) genes [63]. While every Ms4a protein detects specific odors [69], each is likely to play a role in regulating the social acquisition of food preference via olfactory cues [70].

### 3.2. Genetic Regulation in the Vomeronasal System

In the VNS, the VNS sensory neurons (VSNs) are located away from the nasal airstream, and activation of the neurons thus requires a vascular pumping mechanism [71]. This mechanism enables the VNO to take up non-volatile stimuli that are investigated by direct nasal contact [34]. The VSNs are among the most sensitive of mammalian chemoreceptors [72]. In contrast to the main olfactory genes, VNO receptors detect only a limited group of ligands (differential tuning hypothesis [73]). There are 250–300 functional vomeronasal receptor genes [74], in at least three families, including vomeronasal type 1 receptor genes (V1Rs), vomeronasal type 2 receptor genes (V2Rs), and formyl peptide receptor genes (FPRs). As for the ORs in the MOE, the V1Rs and V2Rs encode G- protein-coupled transmembrane proteins [28,39,74].

The main receptor proteins of the VNO consist of the V1R (±150) and V2R (±160) families of vomeronasal receptors [75], each derived from individual genes [29]. The two families of receptors are expressed in anatomically distinct neuronal populations of the VN epithelium [2] that coincide with different zones of G-protein expression (V1R = apical zone of the epithelium, express G_αi2_, dark purple in Figure 1; [28]; V2R = basal zone of the epithelium, express G_0α_, (light purple in Figure 1; [39]), and each VNO neuron expresses only a single receptor protein [75]. In addition, the two classes project to anatomically and functionally separate sub-regions of the AOB, suggesting differential processing of vomeronasal stimuli [76]. V1R-expressing neurons project to the anterior sub-region of the AOB, while V2R-expressing neurons project to the posterior sub-region of the AOB [2]. However, neural projections coming from each of these two regions then project to and overlap at the level of the amygdala, the accessory olfactory tract and the BNST [77].

Unlike OSNs, the VSNs are highly selective for individual molecules [2], although due to their highly diverse nature, V1Rs typically respond to a wide variety of different odor molecules [75,78], and are known to respond to the urinary volatiles 2,3-dehydro-exo-brevicomin (DB) and 2-sec-butyl-4,5-dihydrothiazole (BT, [72]). Both V1Rs and V2Rs are thought to detect olfactory cues that are related to conspecifics [28,31]. For example, 129/SvEv male mice with a cluster of V1R genes genetically deleted show reduced sexual behavior [79]. Interestingly, H2-Mv (a class of major histocompatibility complex (MHC) proteins) is coexpressed in V2Rs in rodents [80], with M10 and M1 family proteins being expressed exclusively in the V2Rs [81]. Indeed, it has been suggested that correct V2R expression relies on the M10s [81]. H2-Mv genes are not randomly expressed, and certain combinations of genes are located with particular V2Rs [82], which could explain an individual’s responsiveness to particular MHC-associated chemosignals [2].

The FPRs are another family of olfactory neurons expressed by localized VNO neurons [63,83]. Interestingly, FPR olfactory expression is restricted to rodents [84], and expression of these receptors occurs in a punctate and monogenic pattern in the VSNs [83], which is characteristic of the transcription of olfactory chemoreceptor genes [85]. Within the vomeronasal (VN) epithelium, Fpr-rs3, -rs4, -rs6 and –rs7 are transcribed by neurons in the apical zone, coincident with V1Rs, while Fpr-rs1 is transcribed by neurons in the basal zone, coincident with V2Rs [83]. It has been suggested that FPRs play a role in the detection of pathogens or pathogenic states [83].

## 4. Olfaction and Paternal Care Behavior: Suggested Genetic Regulation

Numerous candidate genes, many coding for hormone expression, influence paternal care behaviors (e.g., estrogen receptor alpha (ERα) [86]). However, the regulation of paternal care is likely under multisensory control, and olfactory stimuli from pups should be neurally integrated to allow males to recognize offspring [23] and provide paternal care accordingly. The use of odor for distinguishing kin relationships [87,88] and for paternal kin discrimination (e.g., golden hamsters (*Mesocricetus auratus*) [89]) is well documented in rodents. For example, disruption or damage to the OB diminishes paternal care behaviors in biparental male prairie voles [45]. However, several olfactory genes, or genes that regulate olfactory processes, could be involved in the regulation of paternal care behaviors (Table 1).

The MOS likely has less of an influence on the expression of paternal care behaviors since MOE receptor gene sequences are conserved across both paternal and non-paternal vertebrate species [30]. However, VNO receptors detect a limited group of ligands [73], and there is species-specific variation in VNO receptor diversity [30], suggesting that the differential detection and signaling in the VNO could be important for the expression of paternal care behavior in biparental species compared to non-paternal species. However, it is equally plausible that the MOS and VNS work synergistically in identifying, recognizing, and discriminating pup odor cues, and that paternal care is mediated by both systems. Below, I discuss 10 genes that likely work mutually to regulate the expression of paternal care in male rodents.

### 4.1. G_αi2_

VSNs in the apical layer of epithelium in the VNS express *G_αi2_* (Table 1), and these cells have been implicated in moderating pup-directed aggression by detecting pup odor cues, major urinary proteins (MUPs), and odor cues from the facial area, such as exocrine gland-secreted peptide 1 (*ESP1*; Table 1; [90,91,92]). Pup odors activate regions of the OB and AOB (Table 1) that are innervated by *G_αi2_* neurons [58], prompting aggression. However, male mice with deletion of the *G_αi2_* gene were less aggressive towards pups, and showed increased grooming and retrieval of pups (Table 1), most likely because activation in the MPOA was increased in *G_αi2_^-/-^* males [93]. It is likely that *G_αi2_* activates *Trp2* and calcium ion entry downstream of V1R activation (Table 1), moderating aggression [93]. *Gαi2* may also interact with FPRs and ORs in the VNO [83,94] to mediate pup-directed aggression and paternal care (Table 1).

### 4.2. Trp2 (or Trpc2)

In the VNO, activation of vomeronasal GPCRs causes a phospholipase C-dependent cascade [81], which regulates the *Trp2* cation channel [81,95]. Consequently, *Trp2* plays an important role in signal transduction in both V1R and V2R-expressing neurons [75,95]. Furthermore, *Trp2* plays a role in the expression of aggression in males (Table 1), with male *Trp2*^-/-^ mice showing deficiency in social recognition of conspecifics [81], and a reduction in aggression in resident-intruder style tests [95,96]. These studies indicate that aggression requires a functional VNO. While commonly associated with sexual behaviors, *Trp2* could mediate paternal care by reducing aggression in males that might otherwise be directed towards their own pups. Inactivation of the *Trp2* channel does not impair detection of MHC peptides [30], most likely because MCH genes are expressed in a subpopulation of basilar VNS neurons, with M10 in particular being related to the expression of V2Rs [81]. This suggests that males could still identify their own pups via an MHC signature, as sensory neurons respond to MHC peptides in both the VNO and the MOE [97]. If *Trp2* in biparental males is deactivated while cohabiting with a female during the gestation period, or by olfactory cues from the pups themselves, this could cause males to respond paternally rather than aggressively towards their young [22]. Female *Trp2*^-/-^ mice show impaired nest building behavior and time spent with pups [98,99], further suggesting that *Trp2* could also be important for regulating some direct paternal care behaviors as well (Table 1).

### 4.3. CD38

Another gene that may play a role in the expression of paternal care behavior is *CD38*, a transmembrane glycoprotein that catalyzes the formation of calcium ion signaling molecules [100] and cyclic ADP-ribose (Table 1; [101]). *CD38*^-/-^ mice do not show deficits in olfactory-guided foraging or habituation to non-social stimuli, indicating that genetic knockout of this gene does not impair olfactory function per se [100]. *CD38* is implicated in the release of the neuropeptide oxytocin (OT; (Table 1) from hypothalamic neurons [100,102]. OT is involved in sexual, affiliative and parental care behaviors [102,103], and can stimulate the release of prolactin, in concert with arginine vasopressin (AVP [104,105]. Neurons in the MPOA can activate dopaminergic neurons in the VTA, which innervate GABA neurons in the nucleus accumbens (NAcc; (Table 1). OT receptors are also found in the NAcc, and male *CD38*^-/-^ mice show reduced expression of OT in the NAcc [101]. The posterior pituitary secretes OT into the general circulation [100], and increased OT receptor binding in the BNST, lateral septum (LS), lateral amygdala and accessory olfactory nucleus is associated with increased paternal care in male meadow voles (*Microtus pennsylvanicus*) (Table 1; [106]).

Exposure to offspring olfactory cues activates the mitral cells of the OB [107], and increases OT expression in the supraoptic nucleus (SON) of male mandarin voles *Lasiopodomys mandarinus* [108] and in the MPOA of male California mice *Peromyscus californicus* (Table 1; [109]). *CD38^-/-^* fathers show consistently decreased levels of plasma and cerebrospinal OT, and concomitantly a reduction in paternal care (Table 1; [100]). *CD38^-/-^* fathers fail to retrieve pups, and show a reduction in pup grooming, crouching and huddling (Table 1; [101]). Since olfactory function in general is not impaired, genetic knockout of the *CD38* gene indicates an inability to identify odor cues specifically related to pups.

### 4.4. Olfr692

*Olfr692* is a member of the OR gene family that is highly expressed in the basal zone of the VNO (Table 1) of adult male mice [1,110], with expression levels similar to those of *Vmn1r188* and *Vmn2r118* in the V1R and V2R families [1]. An extensive number of *Olfr692*-positive cells occurs in the VNOs of adult rodents, but expression is virtually absent in juveniles [1]. This expression pattern contrasts that of the expression pattern of VR genes, which are first expressed in embryos [28], and the few VNO ORs that are mostly expressed in juveniles [94]. This differential expression of *Olfr692* in adults and juveniles suggests that *Olfr692* may play a role in adult-specific behaviors [1].

*Olfr692*-positive cells in the VNO are activated by odor cues from pups [1]. After exposure to pups, virgin male mice show considerable activation of these cells [1], increasing expression of the immediate early gene *Egr1* (Table 1; [110]). However, activation appears to be dependent on prior social and parenting experience, as males that have sired and cared for pups show low activation of the *Olfr692*-expressing neurons, which could be modulated by endocrine mechanisms [1]. This differential expression of *Olfr692* between fathers and non-fathers suggests that *Olfr692* may mediate aggression and infanticide towards novel pups (Table 1; [22,58]). This could be the neural “switch” that results in infanticidal males becoming paternal [111]. Olfactory cues sensed during active sniffing and investigation of the young [23] could activate or alter the activity of MPOA or BNST neurons (Table 1), leading to this switch in behavior [22,58,112], although the absolute role of *Olfr692* in the expression of paternal care behaviors still requires testing.

### 4.5. MUP Genes

Urinary volatile pheromones are bound to highly polymorphic MUPs [113], a polymorphic group known to be important in chemosensory communication [114], and which are potentially detected by TAARs in the MOE (Table 1; [64]). MUPs are synthesized in the liver, and rodents produce 4-15 MUP variants [114], which may interact directly with the chemosensory receptors to provide a reliable signal of individuality [115]. There are approximately 35 genes in the MUP gene cluster on chromosome 4 [116], and both males and females produce MUPs, indicating that they function as chemical signals for both sexes [114]. While MUPs appear to be principally involved in scent-marking communication [114], MUPs might also function to deliver small semiochemicals to the VNO or MOE [117], thus they may have a similar role to odorant binding proteins [118]. MUPs also act through V2Rs to control interspecies defensive, and intra-species aggressive, behaviors [63], and may also be used for kin discrimination (Table 1; [119]). *MUP3* and *MUP20* elicit aggression in male mice (Table 1; [92]); however, but if males recognize the odor cues from their own offspring, this could potentially deactivate MUP genes, leading to a reduction in aggression and promotion of paternal care.

### 4.6. c-Fos, fosB and CREB

*c-Fos* and *fosB* are immediate early genes (genes that are rapidly expressed in response to a stimulus [120]). The expression of *c-Fos* can be increased by abiotic (e.g., light [121]) or social (e.g., Syrian hamsters [122]) stimuli and, as *fosB* is homologous to *c-Fos*, it follows a similar induction pattern [23]. *fosB* is expressed in several brain regions, including the OB, AOB and PC (Table 1; [123]). Both *c-Fos* and *fosB* are expressed in the MPOA (Table 1; [52,123,124]), particularly in response to pup exposure, which indicates that *c-Fos* and *fosB* neurons are involved in general pup recognition [123,124], regardless of kin relationship. However, the exact neural properties and connections of *c-Fos* positive neurons in the MPOA (Table 1) during active parental care is not well known [23]. Interestingly, while male genetic knockouts for the *fosB* gene showed no impairment in general olfactory discrimination [23], they were less paternal towards pups, and were impaired in their retrieval responses (Table 1; [123]), suggesting that recognition of pups (regardless of kin relationship) specifically is impaired.

Pup-related olfactory stimuli could activate MPOA via activation of brain-derived neurotrophic factor (BDNF), *CD38* or *Trp2* by *G_αi2_*(Table 1), stimulating calcium ion channels [124]. Extracellular signal regulated kinase (ERK) is then phosphorylated in the MPOA neurons (Table 1), inducing transcription of *c-Fos* and *fosB*, which could cause the upregulation of *SPRY1* and *Rad*, leading to increasing paternal care (Table 1; [124]). However, it is possible that, in males, ERK also works in concert with the Ca^2+^/cAMP-responsive element-binding protein (*CREB;* Table 1), as *CREB* increases in the female MPOA following pup exposure [125], and affects maternal behavior [126]. *G_αolf_* [127], *Adyc3* [128], and cyclic nucleotide gated (CNG) cation channels [129] are all enriched in the MOE (Table 1), indicating an important role for cAMP in olfactory signaling [130]. Pup exposure stimulates *CREB*, leading to an increased calcium influx in MPOA neurons that express *Esr1^+^* and *Gal^+^* [131,132], and *Gal^+^* neurons are known to regulate paternal care in mice (Table 1; [22]). Alternately, *CREB* may activate *ERα* [125], which could then affect paternal care (Table 1).

### 4.7. Adyc3

The adenylate cyclase type 3 (*Adcy3*) gene encodes type 3 adenylyl cyclase (AC3 [133]), which is coupled to some odorant receptors [130]. Both *Adyc3* and *G_αolf_* are required for sensory transduction in the MOE (Table 1; [133]). *Adyc3* is also expressed in several brain regions, including the amygdala and MPOA (Table 1; [130]), suggesting a potential role in parental behavior. Female genetic knockouts for *Adyc3* and *G_αolf_* show impaired pup retrieval, nest building and huddling behaviors [68,130]. These responses are likely a consequence of an inability to detect pup odor cues [130]. Similarly, male *Adyc3*^-/-^ males are anosmic, and unable to detect pup odors (Table 1), suggesting that cAMP plays a role in olfactory signaling in males [134]. Interestingly, aggression may also be mediated by *Adyc3*, as female *Adyc3*^-/-^ mice are not aggressive to an intruder that represented a threat to pups, and were not aggressive towards alien pups [130]. It is further possible that motivation to provide paternal care is driven by dopamine through modulation of dopamine type 1 (D1) receptor-dependent cAMP signaling by *G_αolf_* (Table 1; [135]).

### 4.8. PRLR

Prolactin is a gonadotropic hormone secreted by the anterior pituitary [136], but inhibited by dopamine from the hypothalamus (Table 1; [137]). It can cross the blood-brain barrier via a receptor-mediated transport mechanism in the choroid plexus [138], entering the cerebrospinal fluid, and exerting a direct influence in the brain (Table 1; [136,137]). Circulating prolactin increases before parturition in some paternal species [139], and might be critical for organizing neural substrates associated with paternal care behaviors [140].

*PRLR* is an imprinted gene that is expressed at low levels in the OB, and mediates paternal-offspring recognition via olfactory neurogenesis (Table 1; [86]). Mak and Weiss [141] found that neurogenesis under the influence of prolactin signaling increased in in the subventricular zone (SVZ) and dentate gyrus of male house mice (*Mus domesticus*) following interactions with pups (Table 1). Similarly, prolactin receptor mRNA transcript levels increased in the choroid plexus of male Djungarian hamsters (*Phodopus campbelli*) during the early postnatal period [142]. Some of the cells in the mouse SVZ and dentate gyrus matured into olfactory interneurons, and responded preferentially to offspring odors compared to other odor types, indicating a central role of *PRLR* in offspring recognition (Table 1; [141]).

## 5. Conclusions

Pup-related odor cues are critical for the onset and maintenance of mammalian paternal care. However, there are numerous genetic mechanisms underlying the detection, recognition and discrimination of rodent pups, which suggests complex modulation of paternal care behaviors. In this review, I discussed 10 genes that have been implicated in the regulation of paternal care via pup-related olfactory cues in rodents. There are likely many more. That paternal care is likely under multisensory control further complicates our understanding of the direct effects of olfactory genes on the regulation of paternal care behaviors. Since much of our current understanding of the genetic regulation of paternal care via olfaction in rodents comes from studies of laboratory mice, future studies should begin to explore what role, if any, these genes play in the regulation and expression of paternal care in naturally biparental species, such as prairie voles and Djungarian hamsters.

## Figures and Tables

**Table 1:** Genes involved in the regulation and expression of paternal care behaviors in rodents, their theorized olfactory location, functioning associated brain regions and interactions with other genes, proteins or hormones (all references provided in text).

Gene	Location	Associated Brain Regions	Interactions	Effect on Behavior
*G_αi2_*	V1Rs in the apical zone of the VN epithelium	AOB, MPOA, amygdala, BNST, hypothalamus, LPOA, SI, LH, VTA	*Trp2*, FPRs and ORs; *c-Fos, fosB, CREB, ERK. SPRY1, Rad,* MUPs, *ESP1*	Genetic KO = ↓ aggression, ↑ grooming, ↑ retrieval
*Trp2*	V1Rs and V2Rs in the VNO	AOB, MPOA, amygdala, BNST, hypothalamus, LPOA, SI, LH, VTA	*G_αi2_*, *Gal^+^*, *c-Fos, fosB, CREB, ERK. SPRY1, Rad, Esr1^+^*	Genetic KO = ↓ aggression, ↓ recognition, ↓ nest-building, ↓ time with pups
*CD38*	ORs in the MOS	MPOA, BNST, LS, amygdala, AOB, OB, SON, NAcc, olfactory nucleus, PC, orbitofrontal, prefrontal, perirhinal cortex and entorhinal cortices, hippocampus	OT, GABA, c-*Fos*, calcium ion signaling molecules, cyclic ADP-ribose	Genetic KO = ↓ retrieval, ↓ grooming, ↓ huddling
*Olfr692*	ORs in the VNO	AOB, MPOA, amygdala, BNST, hypothalamus, LPOA, SI, LH, VTA	*Egr1, G_αo+_*	↑ expression (virgin ♂s) = ↑ aggression, ? paternal care
MUP genes	TAARs in the MOE; V2Rs in the VNO	OB, AOB, MPOA, amygdala, BNST, hypothalamus, LPOA, SI, LH, VTA, LS, SON, NAcc, olfactory nucleus, PC, orbitofrontal, prefrontal, perirhinal and entorhinal cortices, hippocampus	*G_αi2_*	↑ kin discrimination, ↑ aggression to non-kin
c-*Fos*	ORs in the MOE; VRs in the VNO	OB, AOB, PC, MPOA, amygdala, BNST, hypothalamus, LPOA, SI, LH, VTA, LS, SON, NAcc, olfactory nucleus, orbitofrontal, prefrontal, perirhinal and entorhinal cortices, hippocampus	*fosB, CREB, G_αi2_,* BDNF, *CD38, Trp2,* ERK, *SPRY1, Rad,* *G*_α_*_olf_*, *Adyc3*, CNG, *Esr1^+^*, *Gal^+^*, *ERα*	↑ paternal care
*fosB*	ORs in the MOE; VRs in the VNO	OB, AOB, PC, MPOA, amygdala, BNST, hypothalamus, LPOA, SI, LH, VTA, LS, SON, NAcc, olfactory nucleus, orbitofrontal, prefrontal, perirhinal and entorhinal cortices, hippocampus	*c-Fos, CREB, G_αi2_,* BDNF, *CD38, Trp2,* ERK, *SPRY1, Rad,* *G*_α_*_olf_*, *Adyc3*, CNG, *Esr1^+^*, *Gal^+^*, *ERα*	Genetic KO = ↓ retrieval
*CREB*	ORs in the MOE; VRs in the VNO	OB, AOB, PC, MPOA, amygdala, BNST, hypothalamus, LPOA, SI, LH, VTA, LS, SON, NAcc, olfactory nucleus, orbitofrontal, prefrontal, perirhinal and entorhinal cortices, hippocampus	*c-Fos, fosB, G_αi2_,* BDNF, *CD38, Trp2,* ERK, *SPRY1, Rad,* *G*_α_*_olf_*, *Adyc3*, CNG, *Esr1^+^*, *Gal^+^*, *ERα*	↑ paternal care
*Adyc3*	ORs in the MOE	Amygdala, MPOA, BNST, LS, AOB, OB, SON, NAcc, olfactory nucleus, PC, orbitofrontal, prefrontal, perirhinal and entorhinal cortices, hippocampus	*c-Fos, fosB,CREB,* dopamine, *G*_α_*_olf_*	Genetic KO = ↓ general pup recognition
*PRLR*	ORs in the MOE; V1Rs in the apical epithelium	Choroid plexus, SVZ, dentate gyrus, OB, hippocampus, amygdala, MPOA, BNST, LS, AOB, OB, SON, NAcc, olfactory nucleus, PC, orbitofrontal, prefrontal, perirhinal and entorhinal cortices	Dopamine, AVP	↑ paternal care, ↑ kin pup recognition

V1R: vomeronasal type 1 receptor; V2R: vomeronasal type 2 receptor; OB: main olfactory bulb; AOB: accessory olfactory bulb; MPOA: medial preoptic area; BNST: bed nucleus of the stria terminalis; LPOA: lateral preoptic area; SI: substantia innominate; LH: lateral hypothalamus; KO: knockout; VNO: vomeronasal organ; MOS: main olfactory system; OR: olfactory receptors; OT: oxytocin; MOE: main olfactory epithelium; VTA: ventral tegmental area; SON: supraoptic nucleus; SVZ: subventricular zone; NAcc: nucleus accumbens; FPR: formyl peptide receptor genes; CNG: cyclic nucleotide-gated; BDNF: brain-derived neurotrophic factor; AVP: arginine vasopressin.

## References

[1] Nakahara T.S., Cardozo L.M., Ibarra-Soria X., Bard A.D., Carvalho V.M.A., Trintinalia G.Z., Logan D.W., Papes F. (2016). Detection of pup odors by non-canonical adult vomeronasal neurons expressing an odorant receptor gene is influenced by sex and parenting status. BMC Biol..

[2] Brennan P.A., Keverne E.B. (2004). Something in the air? New insights into mammalian pheromones. Curr. Biol..

[3] Swaney W.T., Curley J.P., Champagne F.A., Keverne E.B. (2008). The paternally expressed Gene *Peg3* regulates sexual experience-dependent preferences for estrous odors. Behav. Neurosci..

[4] Phillips M.L., Tang-Martinez Z. (1998). Parent–offspring discrimination in the prairie vole and the effects of odors and diet. Can. J. Zool..

[5] Been L.E., Petrulis A. (2011). Chemosensory and hormone information are relayed directly between the medial amygdala, posterior bed nucleus of the stria terminalis, and medial preoptic area in male Syrian hamsters. Horm. Behav..

[6] Rymer T.L., Pillay N. (2018). An integrated understanding of paternal care in mammals: Lessons from the rodents. J. Zool..

[7] Schradin C., Anzenberger G. (2001). Costs of infant carrying in common marmosets, *Callithrix jacchus*: An experimental analysis. Anim. Behav..

[8] Campbell J.C., Laugero K.D., Van Westerhuyzen J.A., Hostetler C.M., Cohen J.D., Bales K.L. (2009). Costs of pair-bonding and paternal care in male prairie voles (*Microtus ochrogaster*). Physiol. Behav..

[9] Houston A.I., Székely T., McNamara J.M. (2005). Conflict between parents over care. Trends Ecol. Evol..

[10] Getz L.L., McGuire B. (1993). A comparison of living singly and in male–female pairs in the prairie vole, *Microtus ochrogaster*. Ethology.

[11] Maynard Smith J. (1977). Parental investment: A prospective analysis. Anim. Behav..

[12] Gubernick D.J., Teferi T. (2000). Adaptive significance of male parental care in a monogamous mammal. Proc. Roy. Soc. Lond. B.

[13] Tinbergen N. (1963). On aims and methods of ethology. Z. Tierpsychol..

[14] Runcie M.J. (2000). Biparental care and obligate monogamy in the rock-haunting possum, *Petropseudes dahli*, from tropical Australia. Anim. Behav..

[15] Lee A.W., Brown R.E. (2002). Medial preoptic lesions disrupt parental behavior in both male and female California mice (*Peromyscus californicus*). Behav. Neurosci..

[16] Kohl J., Autry A.E., Dulac C. (2016). The neurobiology of parenting: A neural circuit perspective. Bioessays.

[17] Krettek J.E., Price J.L. (1978). Amygdaloid projections to subcortical structures within the basal forebrain and brainstem in the rat and cat. J. Comp. Neurol..

[18] Simerly R.B., Swanson L.W. (1986). The organization of neural inputs to the medial preoptic nucleus of the rat. J. Comp. Neurol..

[19] Numan M. (1988). Neural basis of maternal behavior in the rat. Psychoneuroendocrinology.

[20] Numan M., Corodimas K.P., Numan M.J., Factor E.M., Piers W.D. (1988). Axon-sparing lesions of the preoptic region and substantia innominata disrupt maternal behavior in rats. Behav. Neurosci..

[21] Tsuneoka Y., Maruyama T., Yoshida S., Nishimori K., Kato T., Numan M., Kuroda K.O. (2013). Functional, anatomical, and neurochemical differentiation of medial preoptic area subregions in relation to maternal behavior in the mouse. J. Comp. Neurol..

[22] Wu Z., Autry A.E., Bergan J.F., Watabe-Uchida M., Dulac C.G. (2014). Galanin neurons in the medial preoptic area govern parental behaviour. Nature.

[23] Kuroda K.O., Tachikawa K., Yoshida S., Tsuneoka Y., Numan M. (2011). Neuromolecular basis of parental behavior in laboratory mice and rats: With special emphasis on technical issues of using mouse genetics. Prog. Neuro-Psychopharmacol. Biol. Psych..

[24] Swanson L.W., Petrovich G.D. (1998). What is the amygdala?. Trends Neurosci..

[25] Scalia F., Winans S.S. (1975). The differential projections of the olfactory bulb and accessory olfactory bulb in mammals. J. Comp. Neurol..

[26] Bargmann C.I. (2006). Comparative chemosensation from receptors to ecology. Nature.

[27] Buck L., Axel R. (1991). A novel multigene family may encode odorant receptors: A molecular basis for odor recognition. Cell.

[28] Dulac C., Axel R. (1995). A novel family of genes encoding putative pheromone receptors in mammals. Cell.

[29] Zhang X., Rodriguez I., Mombaerts P., Firestein S. (2004). Odorant and vomeronasal receptor genes in two mouse genome assemblies. Genomics.

[30] Swaney W.T., Keverne E.B. (2009). The evolution of pheromonal communication. Behav. Brain Res..

[31] Jiao H., Hong W., Nevo E., Li K., Zhao H. (2019). Convergent reduction of *V1R* genes in subterranean rodents. BMC Evol. Biol..

[32] Brechbuhl J., Klaey M., Broillet M.C. (2008). Grueneberg ganglion cells mediate alarm pheromone detection in mice. Science.

[33] Breer H., Fleischer J., Strotmann J. (2006). The sense of smell: Multiple olfactory subsystems. Cell. Mol. Life Sci..

[34] Luo M., Fee M.S., Katz L.C. (2003). Encoding pheromonal signals in the accessory bulb of behaving mice. Science.

[35] Rouquier S., Blancher A., Giorgi D. (2000). The olfactory receptor gene repertoire in primates and mouse: Evidence for reduction of the functional fraction in primates. Proc. Natl. Acad. Sci. USA.

[36] Nagai M.H., Armelin-Correa L.M., Malnic B. (2016). Mongenic and monoallelic expression of odorant receptors. Mol. Pharmacol..

[37] Feinstein P., Bozza T., Rodriguez I., Vassalli A., Mombaerts P. (2004). Axon guidance of mouse olfactory sensory neurons by odorant receptors and the ß2 adrenergic receptor. Cell.

[38] Ressler K.J., Sullivan S.L., Buck L.B. (1994). Information coding in the olfactory system: Evidence for a stereotyped and highly organized epitope map in the olfactory bulb. Cell.

[39] Herrada G., Dulac C. (1997). A novel family of putative pheromone receptors in mammals with a topographically organized and sexually dimorphic distribution. Cell.

[40] Kambere M.B., Lane R.P. (2007). Co-regulation of a large and rapidly evolving repertoire of odorant receptor genes. BMC Neurosc..

[41] Sato T., Hirono J., Hamana H., Ishikawa T., Shimizu A., Takashima I., Kajiwara R., Iijima T. (2008). Architecture of odor information processing in the olfactory system. Anat. Sci. Int..

[42] Haberly L.B. (2001). Parallel-distributed processing in olfactory cortex: New insights from morphological and physiological analysis of neuronal circuitry. Chem. Senses.

[43] Kritzer M. (2004). The distribution of immunoreactivity for intracellular androgen receptors in the cerebral cortex of hormonally intact adult male and female rats: Localization in pyramidal neurons making corticocortical connections. Cereb. Cortex.

[44] Kirkpatrick B., Williams J.R., Slotnick B.M., Carter C.S. (1994). Olfactory bulbectomy decreases social behavior in male prairie voles (*M. ochrogaster*). Physiol. Behav..

[45] Keverne E. (1999). The vomeronasal organ. Science.

[46] Li C.-S., Kaba H., Saito H., Seto K. (1990). Neural mechanisms underlying the action of primer pheromones in mice. Neuroscience.

[47] Halpern M. (1987). The organization and function of the vomeronasal system. Annu. Rev. Neurosci..

[48] Holy T.E., Dulac C., Meister M. (2000). Responses of vomeronasal neurons to natural stimuli. Science.

[49] Grus W.E., Shi P., Zhang Y.-P., Zhang J. (2005). Dramatic variation of the vomeronasal pheromone gene repertoire among five orders of placental and marsupial mammals. Proc. Natl. Acad. Sci. USA.

[50] Beny Y., Kimchi T. (2014). Innate and learned aspects of pheromone-mediated social behaviours. Anim. Behav..

[51] Kevetter G.A., Winans S.S. (1981). Connections of the corticomedial amygdala in the golden hamster. I. Efferents of the “vomeronasal amygdala”. J. Comp. Neurol..

[52] Fernandez-Fewell G.D., Meredith M. (1994). c-Fos expression in vomeronasal pathways of mated or pheromone-stimulated male golden hamsters: Contributions from vomeronasal sensory input and expression related to mating performance. J. Neurosci..

[53] Segovia S., Garcia-Falgueras A., Carrillo B., Collado P., Pinos H., Perez-Laso C., Vinader-Caerols C., Beyer C., Guillamon A. (2006). Sexual dimorphism in the vomeronasal system of the rabbit. Brain Res..

[54] Hines M., Allen L.S., Gorski R.A. (1992). Sex differences in subregions of the medial nucleus of the amygdala and the bed nucleus of the stria terminalis of the rat. Brain Res..

[55] del Abril A., Segovia S., Guillamón A. (1987). The bed nucleus of the stria terminalis in the rat: Regional sex differences controlled by gonadal steroids early after birth. Dev. Brain Res..

[56] Dulac C., O’Connell L.A., Wu Z. (2014). Neural control of maternal and paternal behaviors. Science.

[57] De Vries G.J., Villalba C. (1997). Brain sexual dimorphism and sex differences in parental and other social behaviors. Ann. NY Acad. Sci..

[58] Tachikawa K.S., Yoshihara Y., Kuroda K.O. (2013). Behavioral transition from attack to parenting in male mice: A crucial role of the vomeronasal system. J. Neurosci..

[59] Chess A., Simon I., Cedar H., Axel R. (1994). Allelic inactivation regulates olfactory receptor gene expression. Cell.

[60] Serizawa S., Miyamichi K., Nakatani H., Suzuki M., Saito M., Yoshihara Y., Sakano H. (2003). Negative feedback regulation ensures the one receptor-one olfactory neuron rule in mouse. Science.

[61] Malnic B., Hirono J., Sato T., Buck L.B. (1999). Combinatorial receptor codes for odors. Cell.

[62] Young J.M., Shykind B.M., Lane R.P., Tonnes-Priddy L., Ross J.A., Walker M., Williams E.M., Trask B.J. (2003). Odorant receptor expressed sequence tags demonstrate olfactory expression of over 400 genes, extensive alternate splicing and unequal expression levels. Genome Biol..

[63] Bear D.M., Lassance J.-M., Hoekstra H.E., Datta S.R. (2016). The evolving neural and genetic architecture of vertebrate olfaction. Curr. Biol..

[64] Liberles S.D., Buck L.B. (2006). A second class of chemosensory receptors in the olfactory epithelium. Nature.

[65] Johnson M.A., Tsai L., Roy D.S., Valenzuela D.H., Mosley C., Magklara A., Lomvardas S., Liberles S.D., Barnea G. (2012). Neurons expressing trace amine-associated receptors project to discrete glomeruli and constitute an olfactory subsystem. Proc. Natl. Acad. Sci. USA.

[66] Liberles S.D. (2009). Trace amine-associated receptors are olfactory receptors in vertebrates. Ann. NY Acad. Sci..

[67] Hervé D., Le Moine C., Corvol J.C., Belluscio L., Ledent C., Fienberg A.A., Jaber M., Studler J.M., Girault J.A. (2001). Galpha(olf) levels are regulated by receptor usage and control dopamine and adenosine action in the striatum. J. Neurosci..

[68] Belluscio L., Gold G.H., Nemes A., Axel R. (1998). Mice deficient in G(olf) are anosmic. Neuron.

[69] Greer P.L., Bear D.M., Lassance J.-M., Bloom M.L., Tsukahara T., Pashkovski S.L., Masuda F.K., Nowlan A.C., Kirchner R., Hoekstra H.E. (2016). A family of non-GPCR chemosensors defines an alternative logic for mammalian olfaction. Cell.

[70] Munger S.D., Leinders-Zufall T., McDougall L.M., Cockerham R.E., Schmid A., Wandernoth P., Wennemuth G., Biel M., Zufall F., Kelliher K.R. (2010). An olfactory subsystem that detects carbon disulfide and mediates food-related social learning. Curr. Biol..

[71] Meredith M. (1994). Chronic recording of vomeronasal pump activation in awake behaving hamsters. Physiol. Behav..

[72] Leinders-Zufall T., Lane A.P., Puche A.C., Ma W., Novotny M.V., Shipley M.T., Zufall F. (2000). Ultrasensitive pheromone detection by mammalian vomeronasal neurons. Nature.

[73] Grus W.E., Zhang J. (2008). Distinct evolutionary patterns between chemoreceptors of 2 vertebrate olfactory systems and the differential tuning hypothesis. Mol. Biol. Evol..

[74] Ryba N.J.P., Tirindelli R. (1997). A new multigene family of putative pheromone receptors. Neuron.

[75] Mundy N.I. (2006). Genetic basis of olfactory communication in primates. Am. J. Primatol..

[76] Halpern M., Jia C., Shapiro L.S. (1998). Segregated pathways in the vomeronasal system. Microsc. Res. Techniq..

[77] von Campenhausen H., Mori K. (2000). Convergence of segregated pheromonal pathways from the accessory olfactory bulb to the cortex in the mouse. Eur. J. Neurosci..

[78] Rodriguez I., Del Punta K., Rothman A., Ishii T., Mombaerts P. (2002). Multiple new and isolated families within the mouse superfamily of V1r vomeronasal receptors. Nature Neurosci..

[79] Del Punta K., Leinders-Zufall T., Rodriguez I., Jukam D., Wysocki C.J., Ogawa S., Zufall F., Mombaerts P. (2002). Deficient pheromone responses in mice lacking a cluster of vomeronasal receptor genes. Nature.

[80] Loconto J., Papes F., Chang E., Stowers L., Jones E.P., Takada T., Kumánovics A., Lindahl K.F., Dulac C. (2003). Functional expression of murine V2R pheromone receptors involves selective association with the M10 and M1 families of MHC class Ib molecules. Cell.

[81] Dulac C., Torello A.T. (2003). Molecular detection of pheromone signals in mammals: From genes to behaviour. Nature Rev. Neurosci..

[82] Ishii T., Hirota J., Mombaerts P. (2003). Combinatorial coexpression of neural and immune multigene families in mouse vomeronasal sensory neurons. Curr. Biol..

[83] Rivière S., Challet L., Fluegge D., Spehr M., Rodriguez I. (2009). Formyl peptide receptor-like proteins are a novel family of vomeronasal chemosensors. Nature.

[84] Dietschi Q., Tuberosa J., Rösingh L., Loichot G., Ruedi M., Carleton A., Rodriguez I. (2017). Evolution of immune chemoreceptors into sensors of the outside world. Proc. Natl. Acad. Sci. USA.

[85] Dalton R.P., Lomvardas S. (2015). Chemosensory receptor specificity and regulation. Annu. Rev. Neurosci..

[86] Champagne F.A., Curley J.P., Royle N.J., Smiseth P.T., Kölliker M. (2012). Genetics and epigenetics of parental care. The Evolution of Parental Care.

[87] Schwagmeyer P.L. (1988). Ground squirrel kin recognition abilities: Are there social and life-history correlates?. Behav. Genet..

[88] Widdig A. (2007). Paternal kin discrimination: The evidence and likely mechanisms. Biol. Rev..

[89] Todrank J., Heth G., Johnston R.E. (1998). Kin recognition in golden hamsters: Evidence for kinship odour. Anim. Behav..

[90] Chamero P., Marton T.F., Logan D.W., Flanagan K., Cruz J.R., Saghatelian A., Cravatt B.F., Stowers L. (2007). Identification of protein pheromones that promote aggressive behaviour. Nature.

[91] Chamero P., Katsoulidou V., Hendrix P., Bufe R., Roberts R., Matsunami H., Abramowitz J., Birnbaumer L., Zufall F., Leinders-Zufall T. (2011). G protein Gαo is essential for vomeronasal function and aggressive behavior in mice. Proc. Natl. Acad. Sci. USA.

[92] Kaur A.W., Ackels T., Kuo T.-H., Cichy A., Dey S., Hays C., Kateri M., Logan D.W., Marton T.F., Spehr M. (2014). Murine pheromone proteins constitute a context-dependent combinatorial code governing multiple social behaviors. Cell.

[93] Trouillet A.-C., Keller M., Weiss J., Leinders-Zufall T., Birnbaumer L., Zufall F., Chamero P. (2019). Central role of G protein Gαi2 and Gαi2^+^ vomeronasal neurons in balancing territorial and infant-directed aggression of male mice. Proc. Natl. Acad. Sci. USA.

[94] Lévai O., Feistel T., Breer H., Strotman J. (2006). Cells in the vomeronasal organ express odorant receptors but project to the accessory olfactory bulb. J. Comp. Neurol..

[95] Leypold B.G., Yu C.R., Leinders-Zufall T., Kim M.M., Zufall F., Axel R. (2002). Altered sexual and social behaviors in trp2 mutant mice. Proc. Natl. Acad. Sci. USA.

[96] Stowers L., Holy T.E., Meister M., Dulac C., Koentges G. (2002). Loss of sex discrimination and male-male aggression in mice deficient for TRP2. Science.

[97] Spehr M., Kelliher K.R., Li X.-H., Boehm T., Leinders-Zufall T., Zufall F. (2006). Essential role of the main olfactory system in social recognition of major histocompatibility complex peptide ligands. J. Neurosci..

[98] Hasen N.S., Gammie S.C. (2009). *Trpc2* gene impacts on maternal aggression, accessory olfactory bulb anatomy and brain activity. Genes Brain Behav..

[99] Kimchi T., Xu J., Dulac C. (2007). A functional circuit underlying male sexual behvior in the female mouse brain. Nature.

[100] Jin D., Liu H.-X., Hirai H., Torashima T., Nagai T., Lopatina O., Shnayder N.A., Yamada K., Noda M., Seike T. (2007). CD38 is critical for social behaviour by regulating oxytocin secretion. Nature.

[101] Akther S., Korshnova N., Zhong J., Liang M., Cherepanov S.M., Lopatina O., Komleva Y.K., Salmina A.B., Nishimura T., Fakhrul A.A.K.M. (2013). CD38 in the nucleus accumbens and oxytocin are related to paternal behavior in mice. Mol. Brain.

[102] Grigor’eva M.E., Golubeva M.G. (2010). Oxytocin: Structure, synthesis, receptors, and basic effects. Neurochem. J..

[103] Marsh A.A., Yu H.H., Pine D.S., Gorodetsky E.K., Goldman D., Blair R.J.R. (2012). The influence of oxytocin administration on responses to infant faces and potential moderation by *OXTR* genotype. Psychopharmacology.

[104] Barberis C., Tribollet E. (1996). Vasopressin and oxytocin receptors in the central nervous system. Crit. Rev. Neurobiol..

[105] Cho M.M., DeVries A.C., Williams J.R., Carter C.S. (1999). The effects of oxytocin and vasopressin on partner preferences in male and female prairie voles (*Microtus ochrogaster*). Behav. Neurosci..

[106] Parker K.J., Kinney L.F., Phillips K.M., Lee T.M. (2001). Paternal behavior is associated with central neurohormone receptor binding patterns in meadow voles (*Microtus pennsylvanicus*). Behav. Neurosci..

[107] Lopatina O., Inzhutova A., Pichugina Y.A., Okamato H., Salmina A.B., Higashida H. (2011). Reproductive experience affects parental retrieval behaviour associated with increased plasma oxytocin levels in wild-type and *CD38*-knockout mice. J. Neuroendocrinol..

[108] Song Z., Tai F., Yu C., Wu R., Zhang X., Broders H., He F., Guo R. (2010). Sexual or paternal experiences alter alloparental behavior and the central expression of ERα and OT in male mandarin voles (*Microtus mandarinus*). Behav. Brain Res..

[109] Lambert K.G., Franssen C.L., Hampton J.E., Rzucidlo A.M., Hyer M.M., True M., Kaufman C., Bardi M. (2013). Modeling paternal attentiveness: Distressed pups evoke differential neurobiological and behavioral responses in paternal and nonpaternal mice. Neuroscience.

[110] Horrell N.D., Hickmott P.W., Saltzman W. (2019). Neural regulation of paternal behaviour in mammals: Sensory, neuroendocrine, and experiential influences on the paternal brain. Curr. Top. Behav. Neurosci..

[111] Svare B., Mann M. (1981). Infanticide: Genetic, developmental and hormonal influences in mice. Physiol. Behav..

[112] Tsuneoka Y., Tokita K., Yoshihara C., Amano T., Esposito G., Huang A.J., Yu L.M., Odaka Y., Shinozuka K., McHugh T.J. (2015). Distinct preoptic-BST nuclei dissociate paternal and infanticidal behavior in mice. EMBO J..

[113] Böcskei Z., Groom C.R., Flower D.R., Wright C.E., Phillips S.E.V., Cavaggioni A., Findlay J.B.C., North A.C.T. (1992). Pheromone binding to two rodent urinary proteins revealed by X-ray crystallography. Nature.

[114] Beynon R.J., Hurst J.L. (2003). Multiple roles of major urinary proteins in the house mouse, *Mus domesticus*. Biochem. Soc. Trans..

[115] Flower D.R. (1996). The lipocalin protein family: Structure and function. Biochem. J..

[116] Al-Shawi R., Ghazal P., Clark A.J., Bishop J.O. (1989). Intraspecific evolution of a gene family coding for urinary proteins. J. Mol. Evol..

[117] Utsumi M., Ohno K., Kawasaki Y., Tamura M., Kubo T., Tohyama M. (1999). 1999. Expression of major urinary protein genes in the nasal glands associated with general olfaction. J. Neurobiol..

[118] Cavaggioni A., Mucignat-Caretta C. (2000). Major urinary proteins, α_2U_-globulins and aphrodisin. Biochim. Biophys. Acta.

[119] Busquet N., Baudoin C. (2005). Odour similarities as a basis for discriminating degrees of kinship in rodents: Evidence from *Mus spicilegus*. Anim. Behav..

[120] Jenkins R., Tetzlaff W., Hunt S.P. (1993). Differential expression of immediate early genes in rubrospinal neurons following axotomy in rat. Eur. J. Neurosci..

[121] Moffatt C.A., Ball G.F., Nelson R.J. (1995). The effects of photoperiod on olfactory c-*fos* expression in prairie voles, *Microtus ochrogaster*. Brain Res..

[122] Fiber J.M., Adames P., Swann J.M. (1993). Pheromones induce c-*fos* in limbic areas regulating male hamster mating behaviour. NeuroReport.

[123] Brown J.R., Ye H., Bronson R.T., Dikkes P., Greenberg M.E. (1996). A defect in nurturing in mice lacking the immediate early gene *fosB*. Cell.

[124] Kuroda K.O., Meaney M.J., Uetani N., Fortin Y., Ponton A., Kato T. (2007). ERK-FosB signaling in dorsal MPOA neurons plays a major role in the initiation of parental behavior in mice. Mol. Cell. Neurosci..

[125] Stolzenberg D.S., Mayer H.S. (2019). Experience-dependent mechanisms in the regulation of parental care. Front. Neuroendocrinol..

[126] Jin S.-H., Blendy J.A., Thomas S.A. (2005). Cyclic AMP response element-binding protein is required for normal maternal nurturing behavior. Neuroscience.

[127] Jones D.T., Reed R.R. (1989). G_olf_: An olfactory neuron specific-G protein involved in odorant signal transduction. Science.

[128] Bakalyar H.A., Reed R.R. (1990). Identification of a specialized adenylyl cyclase that may mediate odorant detection. Science.

[129] Nakamura T., Gold G.H. (1987). A cyclic nucleotide-gated conductance in olfactory receptor cilia. Nature.

[130] Wang Z., Storm D.R. (2011). Maternal behavior is impaired in female mice lacking type 3 adenylyl cyclase. Neuropsychopharmacology.

[131] Kohl J., Babayan B.M., Rubinstein N.D., Autry A.E., Marin-Rodriguez B., Kapoor V., Miyamishi K., Zweifel L.S., Luo L., Uchida N. (2018). Functional circuit architecture underlying parental behaviour. Nature.

[132] Fang Y.-Y., Yamaguchi T., Song S.C., Tritsch N.X., Lin D. (2018). A hypothalamic midbrain pathway essential for driving maternal behaviors. Neuron.

[133] Kuroda K.O., Tsuneoka Y. (2013). Assessing postpartum maternal care, alloparental behavior, and infanticide in mice: With notes on chemosensory influences. Methods Mol. Biol..

[134] Wong S.T., Trinh K., Hacker B., Chan G.C., Lowe G., Gaggar A., Xia Z., Gold G.H., Storm D.R. (2000). Disruption of the type III adenylyl cyclase gene leads to peripheral and behavioral anosmia in transgenic mice. Neuron.

[135] Hervé D. (2011). Identification of a specific assembly of the G protein Golf as a critical and regulated module of dopamine and adenosine-activated cAMP pathways in the striatum. Front. Neuroanat..

[136] Saltzman W., Maestripieri D. (2011). The neuroendocrinology of primate maternal behavior. Prog. Neuro-Psychopharmacol. Biol. Psych..

[137] Grattan D.R., Kokay I.C. (2008). Prolactin: A pleiotropic neuroendocrine hormone. J. Neuroendocrinol..

[138] Walsh R.J., Slaby F.J., Posner B.I. (1987). A receptor-mediated mechanism for the transport of prolactin from blood to cerebrospinal fluid. Endocrinology.

[139] Brown R.E., Murdoch T., Murphy P.R., Moger W.H. (1995). Hormonal responses of male gerbils to stimuli from their mate and pups. Horm. Behav..

[140] Schradin C., Anzenberger G. (1999). Prolactin, the hormone of paternity. News Physiol. Sci..

[141] Mak G.K., Weiss S. (2010). Paternal recognition of adult offspring mediated by newly generated CNS neurons. Nature Neurosci..

[142] Ma E., Lau J., Grattan D.R., Lovejoy D.A., Wynne-Edwards K.E. (2005). Male and female prolactin receptor mRNA expression in the brain of a biparental and a uniparental hamster, *Phodopus*, before and after the birth of a litter. J. Neuroendocrinol..

